# Preclinical Evidence for the Efficacy of Ischemic Postconditioning against Renal Ischemia-Reperfusion Injury, a Systematic Review and Meta-Analysis

**DOI:** 10.1371/journal.pone.0150863

**Published:** 2016-03-10

**Authors:** Simone J. Jonker, Theo P. Menting, Michiel C. Warlé, Merel Ritskes-Hoitinga, Kimberley E. Wever

**Affiliations:** 1 SYstematic Review Centre for Laboratory animal Experimentation (SYRCLE), Radboud university medical center, Nijmegen, The Netherlands; 2 Department of surgery, Radboud university medical center, Nijmegen, The Netherlands; University of Sao Paulo Medical School, BRAZIL

## Abstract

**Background:**

Renal ischemia-reperfusion injury (IRI) is a major cause of kidney damage after *e*.*g*. renal surgery and transplantation. Ischemic postconditioning (IPoC) is a promising treatment strategy for renal IRI, but early clinical trials have not yet replicated the promising results found in animal studies.

**Method:**

We present a systematic review, quality assessment and meta-analysis of the preclinical evidence for renal IPoC, and identify factors which modify its efficacy.

**Results:**

We identified 39 publications studying >250 control animals undergoing renal IRI only and >290 animals undergoing renal IRI and IPoC. Healthy, male rats undergoing warm ischemia were used in the vast majority of studies. Four studies applied remote IPoC, all others used local IPoC. Meta-analysis showed that both local and remote IPoC ameliorated renal damage after IRI for the outcome measures serum creatinine, blood urea nitrogen and renal histology. Subgroup analysis indicated that IPoC efficacy increased with the duration of index ischemia. Measures to reduce bias were insufficiently reported.

**Conclusion:**

High efficacy of IPoC is observed in animal models, but factors pertaining to the internal and external validity of these studies may hamper the translation of IPoC to the clinical setting. The external validity of future animal studies should be increased by including females, comorbid animals, and transplantation models, in order to better inform clinical trial design. The severity of renal damage should be taken into account in the design and analysis of future clinical trials.

## Introduction

Renal ischemia and reperfusion injury (IRI) is a major cause of acute kidney injury (AKI) after *e*.*g*. renal surgery, coronary artery bypass grafting and abdominal aortic aneurysm repair, which results in increased morbidity and mortality[1]. Renal IRI is also considered an important cause of delayed graft function after renal transplantation and is associated with prolonged hospital stay and acute rejection[2,3].

Ischemic postconditioning (IPoC) is a protective strategy in which (repeated) brief, intermittent periods of ischemia and reperfusion are applied in the early phase of reperfusion after a prolonged ischemic episode. Since its discovery in 2003 in the dog heart[4], IPoC has been shown to attenuate IRI in various organs and a variety of animal species, and is effective when applied to either the target organ, or a remote organ or tissue[5,6]. Thus, IPoC poses a promising treatment strategy for IRI in patients.

Following the promising results obtained in animal studies, the feasibility and efficacy of renal IPoC in patients has been investigated in two clinical trials[7,8]. Although application of local IPoC seemed feasible and safe in patients undergoing donation-after-circulatory-death kidney transplantation, it had no effect on delayed graft function incidence or renal function in a paired kidney analysis[7]. Remote IPoC (RIPoC) appeared to hasten the early recovery of graft function in patients undergoing living donor kidney transplantation, but did not affect graft function >24 hours post-operatively[8]. In addition, clinical trials investigating the effect of IPoC on the myocardium have also yielded conflicting results (reviewed in[9,10]). Thus, the question arises why the replication of the promising results found in animals has been limited in patients, and how the translation of IPoC from animal studies to patients may be improved.

Previously, meta-analysis and systematic review of preclinical studies have proven useful in optimizing the design of both preclinical and clinical studies[11–13]. Although an overview of experimental studies in this field exists[14], a systematic review of the preclinical evidence for renal IPoC is lacking. It remains unclear if and how factors pertaining to the IPoC protocol (*e*.*g*. timing and duration) and the animals under investigation (*e*.*g*. sex, comorbidities) influence IPoC efficacy. As a result, the IPoC stimulus could have been suboptimal or incorrectly applied in clinical trials, or unsuitable for the patient population. We therefore conducted a systematic review and meta-analysis of evidence on the protective effect of IPoC in animal models of renal IRI. This approach allowed us to analyze the influence of variables such as IPoC timing, IPoC duration, sex and comorbidity on treatment efficacy. We also assessed the extent to which the preclinical data might be at risk of bias, either through publication bias, or through factors relating to experimental design.

## Materials and Methods

For an extended version, see S1 Text. The review methodology was predefined and documented in a protocol[15], published online on February 12^th^ 2015. The review question was: what is the effect of local or remote IPoC on renal function in animal models of renal IRI?

### Amendments to the review protocol

After study selection, we found that the timing and duration of the IPoC protocol depended strongly on the site of postconditioning. We therefore decided to perform separate meta-analyses of studies using local, remote, and local+remote postconditioning, to avoid collinearity.

For serum creatinine and blood urea nitrogen (BUN), all data could be expressed in the same unit of measurement, but differences in baseline measurements between studies were observed. We therefore performed meta-analysis of the normalised mean difference (NMD) instead of the standardized mean difference (SMD). For renal histology, we expressed all scores as a percentage on the grading scale used, and performed meta-analysis of the mean difference (MD), instead of the SMD. This allowed us to include studies reporting the histology score as a percentage on the grading scale used.

### Study identification

A systematic, computerized search in the databases Medline (via PubMed) and EMBASE (S1 Table) was performed on February 4^th^ 2015, using the search components ‘kidney’, ‘ischemic postconditioning’ and an animal search filter for either PubMed[16] or EMBASE[17]. To identify additional relevant studies, the reference lists of included studies and relevant reviews were hand searched. No language restrictions were applied.

### Selection of studies

After removal of duplicates, all references were screened for inclusion based on their title and abstract. The following inclusion criteria were applied: the study 1) is an original article presenting unique data with a control group, 2) is performed *in vivo* in animals with or without comorbidities, but without genetic modifications, 3) reports on renal ischemia-reperfusion injury and outcome measures related to kidney injury or function, and 4) examined the effect of remote and/or local ischemic postconditioning. Subsequently, the full-text manuscripts of eligible studies were reviewed for inclusion. Studies involving co-medication other than anaesthetics or analgesics, or a co-intervention other than collateral nephrectomy were excluded. Studies performed in a renal transplantation model were excluded from the present dataset, but labelled for future reference. In both phases, references were independently assessed for inclusion by two reviewers (KW and SJ).

### Study characteristics and data extraction

Study characteristics were extracted by one reviewer (SJ) and checked for inconsistencies by a second reviewer (TM). We selected the following outcome measures for analysis: serum creatinine, BUN and renal histology scores (Jablonski[18] or comparable). Data was collected as mean and standard deviation (SD). For serum creatinine and BUN, all data was recalculated to the same unit of measurement (respectively umol/L and mmol/L). For renal histology, scores were expressed as a percentage on the grading scale used. If an outcome was measured at several time-points, data was extracted for the time-point of greatest efficacy. If a study reported data from several experimental groups, it was extracted as separate comparisons and the number of animals in the control group was corrected (number of animals divided by number of comparisons).

### Risk of bias and study quality

Two reviewers (SJ and TM) independently assessed the risk of bias and study quality of each included study. In case of discrepancies, consensus was reached by discussion with a third reviewer (KW). Risk of bias was assessed using SYRCLE’s Risk of Bias tool[19]. Reporting bias (item #9) was not assessed, since none of the studies reported the use of a study protocol predefining primary and secondary outcomes. When assessing selection bias, groups within a study were considered similar at baseline if sex and baseline serum creatinine did not significantly differ between groups (or, if baseline creatinine was unavailable, body weight). To assess whether studies were free of other risks of bias, addition of animals to groups during the experiment and a possible conflict of interest were taken into account. We also assessed reporting of the following study quality items: any randomization, any blinding, regulation of body temperature within 3°C variation and sample size calculation.

### Data analysis

Data was analyzed using Stata/SE (StataCorp, Texas, USA). For the outcome measures serum creatinine and BUN, meta-analysis was performed on the NMD, which allows us to correct for baseline kidney injury by relating the magnitude of the effect of treatment to a baseline measured in untreated animals[20]. For histology, the MD was used. A random effects model was used to account for expected between-study heterogeneity. To assess heterogeneity, the I^2^ and adjusted R^2^ statistics were determined. To examine potential sources of heterogeneity, predefined subgroup analyses were performed on subgroups containing data from at least three studies. For the duration of IPoC ischemia, studies were categorized using increments of 0.7 log, which resulted in categories of 26–125, 126–630 and 631–3162 seconds of ischemia. For the duration of index ischemia, studies were categorized using increments of 15 minutes, resulting in categories of 16–30, 31–45, 46–60, 61–75 (no studies) and 76–90 minutes. Differences between subgroups were determined by calculating the difference in NMD and MD respectively and the 95% confidence intervals (CI) of the difference. Results are reported as a NMD or MD [95%-CI], unless stated otherwise. For each outcome measure, the significance level for subgroup analyses was adjusted for the number of analyses using the Bonferroni-Holm method[21].

Publication bias was assessed for each outcome measure by visual evaluation of funnel plots, Duval and Tweedie’s trim and fill analysis and by performing Egger’s test for small study effects. Sensitivity analyses were carried out for creatinine and BUN using a fixed time point of outcome assessment (24 hrs). For histology, a sensitivity analysis was performed using only Jablonski histology scores.

## Results

### Study identification and selection

**Fig 1:**
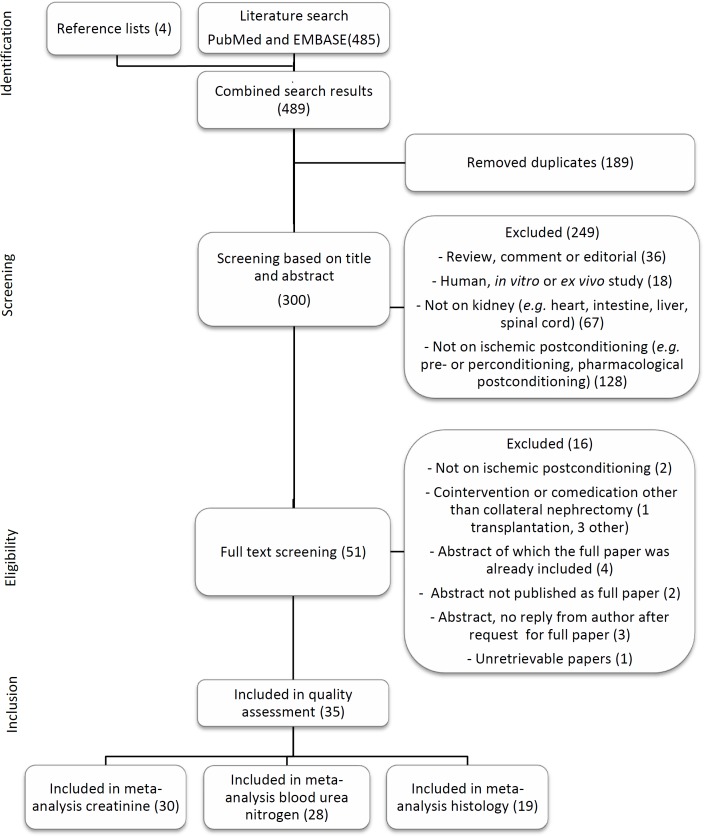
Flow chart of study selection. The number of studies in each phase are shown between brackets.

A flow chart of the study selection process is shown in Fig 1. The computerized search retrieved 213 references from PubMed and 272 from EMBASE. Four additional references were added after hand searching reference lists of included studies and relevant reviews. After duplicate removal, 300 references were screened based on title and abstract and 51 studies continued to the eligibility phase. Two letters to the editor[22,23] were included since they presented unique data and sufficient methodological detail. One study investigating IPoC in a canine model of renal transplantation with cold ischemia was excluded, because of the differences in pathophysiology compared to warm ischemia. Finally, 35 studies were included in the risk of bias assessment, all but one of which reported on one or more of the selected outcome measures.

### Study characteristics

The study characteristics are summarized in Table 1. Out of the 35 included studies, 31 were performed in rats, one in dogs and three in mice. Male animals were used in all but three studies. There were only three studies investigating the effect of RIPoC, all of which used the hind limb as remote tissue. The most commonly used durations of index ischemia were 45 and 60 min. The IPoC protocol varied between studies, however, local application of 6 cycles of 10/10 seconds of reperfusion/ischemia was most commonly used.

**Table 1:** Study characteristics. Duration of index ischemia is given in minutes, IPoC protocol timing and duration in seconds, time of outcome measurement in hours.

Study	Species/ strain	Sex	Index isch	# IPoC cycles	IPoC isch	IPoC rep	IPoC delay	Coll ntx?	Site of IPoC	Time of OM (hrs)	OM
Chen 2008 [41]	R/W	M	45	6	10	10	0	Y	LIPoC	24/48/72	Cr, BUN, H(J)
Chen 2011 [42]	R/SD	M	60	6	10	10	0	Y	LIPoC	4	Cr, BUN, H(D)
Chen 2014 [43]	R/W	M	45	6	10	10	0	Y	LIPoC	24	Cr, BUN, H(J)
Chen 2015 [44]	R/W	M	45	6	10	10	0	Y	LIPoC	24	Cr, BUN, H(J)
Eldaif 2010 [45]	R/SD	M	45	4	45	45	0	Y	LIPoC	24	Cr, BUN, H(O)
Fan 2009 [46]	R/SD	M	60	6	10	10	0	?	LIPoC	6	Cr, BUN, H(D)
Guo 2014 [47]	R/SD	M	45	3	10	10	0	N	LIPoC	0/1/3/6/12/24/48	Cr, BUN
Ji 2012 [48]	R/SD	M	45	6	10	10	0	Y	LIPoC	6/12/24/48/72	Cr, BUN, H(D)
					15	15					
Jiang 2010 [49]	D	M	60	6	30	30	0	Y	LIPoC	72	Cr, BUN, H(J)
					60	60					
Jiang 2014 [50]	R/SD	M	60	4	300	300	0	Y	RIPoC^†^	24	Cr, BUN, H(J)
Kadkhodaee 2011 [22]	R/SD	M	45	4	300	300	0	Y	RIPoC^†^	24	Cr, BUN
Kadkhodaee 2014 [51]	R/SD	M	45	4	10/300	10/300	0	Y	LIPoC/RIPoC^†^	24	Cr, BUN, H(D)
Lemoine 2015 [52]	M/C57BL6	M	30	3	30	30	0	Y	LIPoC	24	Cr, BUN, H(O)
Li 2010 [53]	R/SD	M	45	10	20	20	0	N	LIPoC	24	Cr, BUN, H(J)
Li 2012 [54]	R/SD	F	45	10	20	20	0	Y	LIPoC	1/3/6/12/24	Cr, BUN
Liu 2007 [55]	R/W	M	45	6	10	10	0	Y	LIPoC	24	Cr, BUN, H(J)
Mahfoudh-Boussaid 2012 [24]	R/W	M	60	6	10	10	0	N	LIPoC	2	Cr, H(J)
Mahmoudi 2014 [35]	R/SD	m/f	45	4	10	10	0	Y	LIPoC	24	Cr, BUN, H(O)
Miklós 2012 [26]	R/W	M	45	4	15	15	0	N	LIPoC	2	Cr, BUN, H(J)
Serviddio 2008 [27]	R/W	M	90	3	300	180-360-720*	0	Y	LIPoC	0/0.6/24/48	Cr, BUN, H(O)
Shokeir 2012 [56]	R/SD	M	45	3	300	180-360-720*	0	Y	LIPoC	2/24/48	Cr, BUN, H(O)
Shokeir 2014 [57]	R/SD	M	45	3	300	180-360-720*	0	Y	LIPoC	2/24/48	Cr, BUN
Szwarc 2007 [58]	M/Swiss	F	30	3	30	30	0	Y	LIPoC	0–192	Cr
Tan 2013 [59]	R/SD	M	45	3	30	30	420	Y	LIPoC	1/48/168	Cr, H(D)
Tang 2008 [60]	R/W	M	60	6	10	10	0	Y	LIPoC	24	Other
Tao 2012 [61]	R/SD	m	45	6	10	10	0	Y	LIPoC	24	Cr, BUN, H(D)
Wang 2010 [62]	R/W	m	60	6	10	10	0	Y	LIPoC	24	Cr, BUN, H(J)
Weng 2012 [28]	R/SD	m	45	6	10	10	0	Y	LIPoC	2016	Cr, BUN, H(O)
				6	8	8			LIPoC		
Wever 2012 [23]	R/SD	m	25	3	300	300	0	Y	RIPoC^†^	48	Cr, BUN, H(O)
				9	308	308			Both^†^		
Xia 2014 [25]	R/SD	m	60	6	10	10	0	Y	LIPoC	1/3/6/24	Cr, BUN, H(O)
Yun 2009 A [63]	R/SD	m	45	6	10	10	0	Y	LIPoC	1/3/6/12/24	Cr, BUN, H(J)
Yun 2009 B [64]	R/SD	m	45	6	10	10	0	Y	LIPoC	24	Cr, BUN, H(J)
Zhang 2011 [65]	R/SD	m	45	3	10	10	0	N	LIPoC	6	Cr, BUN, H(D)
Zhu 2008 [66]	R/W	m	60	6	10	10	0	Y	LIPoC	24	H(D)
Zhuang 2009 [67]	M/C57	m	26	3	30	30	0/600	Y	LIPoC	48	Cr, BUN, H(O)

*the duration of reperfusion elongated in each cycle

†remote tissue was the hind limb. Index isch = index ischemia in minutes, IPoC isch = duration of ischemic phase in IPoC protocol, IPoC rep = duration of reperfusion phase in IPoC protocol, IPoC delay = delay between end of index ischemia and start of IPoC, Coll ntx = collateral nephrectomy, OM = outcome measure, R = rat, D = dog, M = mouse, SD = Sprague-Dawley, W = Wistar, m = male, f = female, Y = yes, N = no,? = unknown, LIPoC = local ischemic postconditioning, RIPoC = remote ischemic postonditioning, Cr = serum creatinine, BUN = blood urea nitrogen, H(J) = renal histology assessed by Jablonski score, H(O) = renal histology assessed by other scoring system, H(D) = descriptive reporting of renal histology.

### Risk of bias and study quality

**Fig 2:**
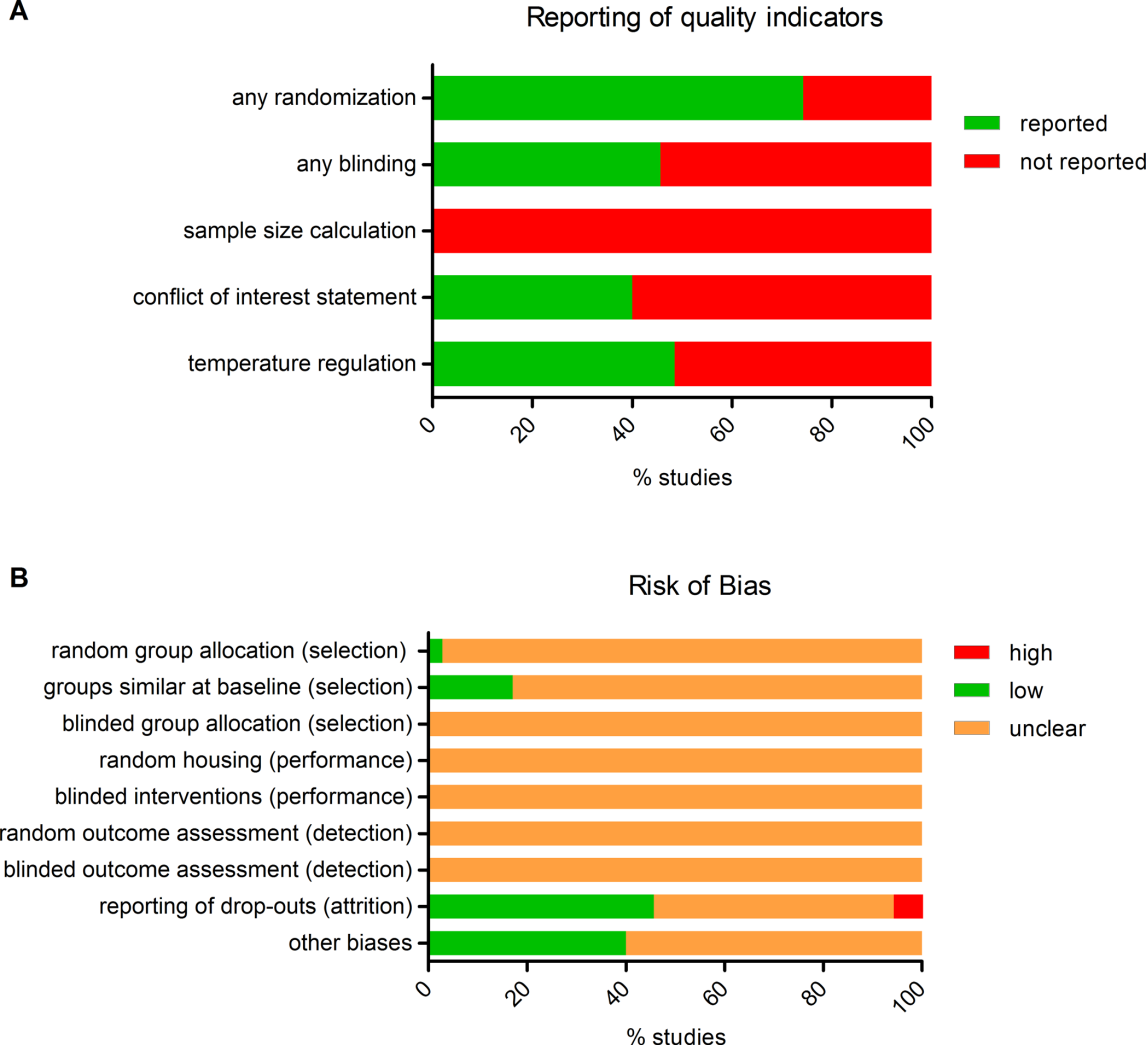
Risk of bias and study quality assessment. **Top**: Reporting of five key study quality indicators was found to be poor in many cases. **Bottom**: Using SYRCLE's risk of bias tool, the risk of selection, performance, detection, attrition and other biases was assessed. Lack of (adequate) reporting of measures to reduce bias resulted in a high percentage of unclear risk of bias for most items.

The results of the study quality and risk of bias assessment are shown in Fig 2 and S2 Table. Randomization and blinding are essential measures to reduce bias, but are infrequently reported. Seventy-four percent of the included studies reported random allocation of the animals, however, only one study adequately specified the method of randomisation. Studies that reported blinding (46%), only did so for the outcome assessment of histology. None of the studies reported a sample size calculation. As a consequence of insufficient reporting, the risk of bias was unclear for most items of the risk of bias tool.

### Meta-analyses

Studies investigating local, remote, or local+remote IPoC were analyzed separately. Only the local IPoC group contained enough studies to perform subgroup analysis for any of the outcome measures. One study reporting creatinine clearance[24] was excluded from analysis because serum creatinine data could not be obtained. Data from two studies[25,26] was excluded because serum creatinine or BUN levels were the same in the experimental group and the sham group, indicating that the experimental group did not sustain a sufficient amount of renal IRI. For renal histology, two studies were excluded due to incomplete outcome data[26]^,^ [27].

#### Serum creatinine

**Fig 3:**
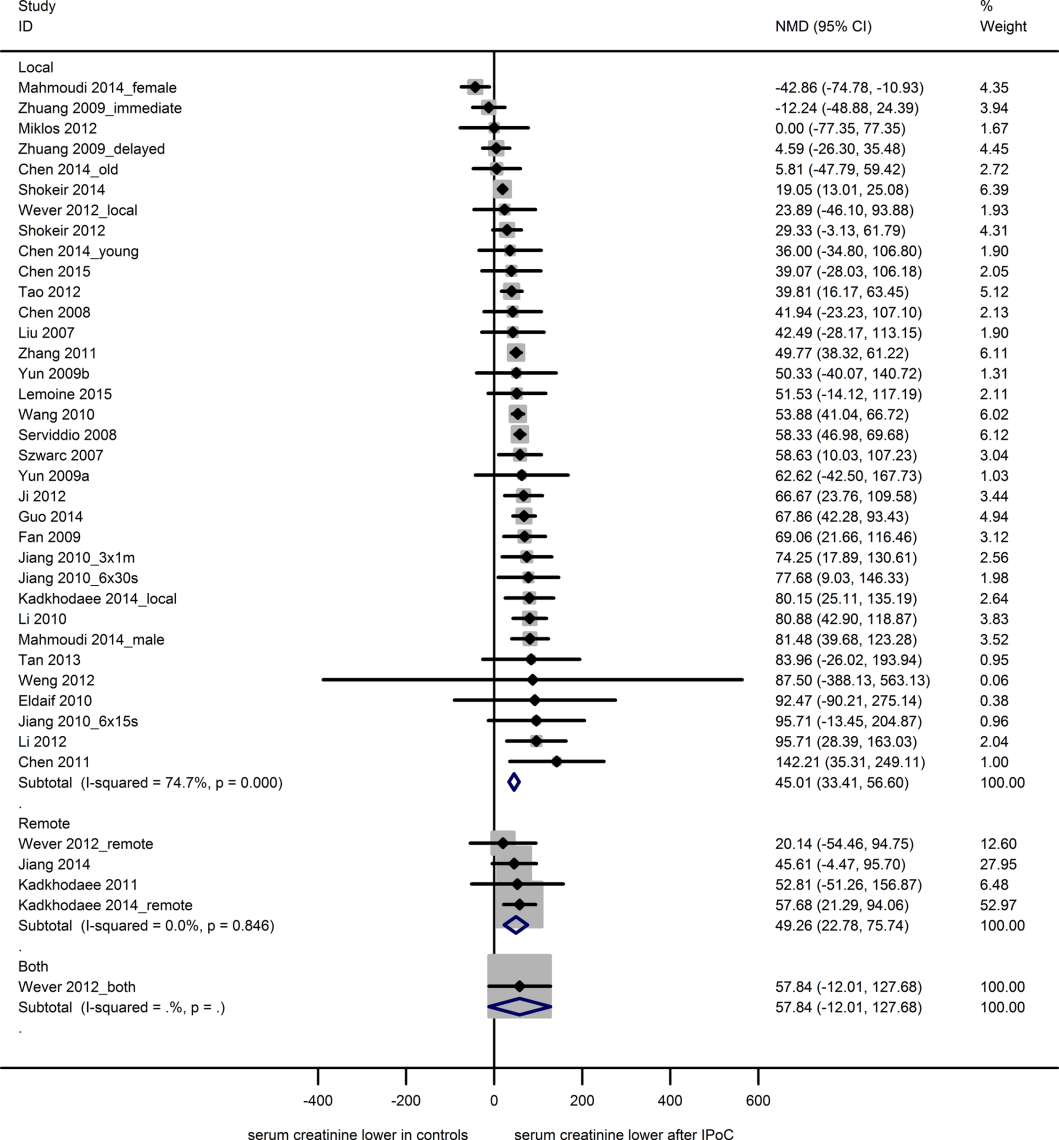
Meta-analysis creatinine. The summary effects show a decrease in serum creatinine after local or remote IPoC. One study investigating the combination of local and remote IPoC showed no effect. Data are presented as NMD and 95% CI. Within subgroup weights from random effects analysis are shown.

Thirty-one studies reported serum creatinine data from 39 experiments, using 258 sham animals, 247 control animals undergoing renal IRI only and 298 experimental animals undergoing both IRI and IPoC. Both the control and experimental groups contained 3 to 12 animals (median n = 8). The IRI-induced rise in serum creatinine was reduced by both LIPoC (34 experiments; NMD 45.0 [33.4, 56.6]) and RIPoC (4 experiments; NMD 49.3 [22.8, 75.7]; Fig 3). One study investigating the combination of LIPoC and RIPoC showed no effect (NMD 57.84 [-12.0, 127.7]).

Subgroup analysis results for the LIPoC studies are shown in S3 Table. LIPoC had a beneficial effect on creatinine in all subgroups, except for mouse, female, 4 cycles of LIPoC and 16–30 minutes of index ischemia. Overall heterogeneity was high (I^2^ 74.7%), but none of the subgroup variables accounted for a significant proportion of the observed heterogeneity.

#### BUN

**Fig 4:**
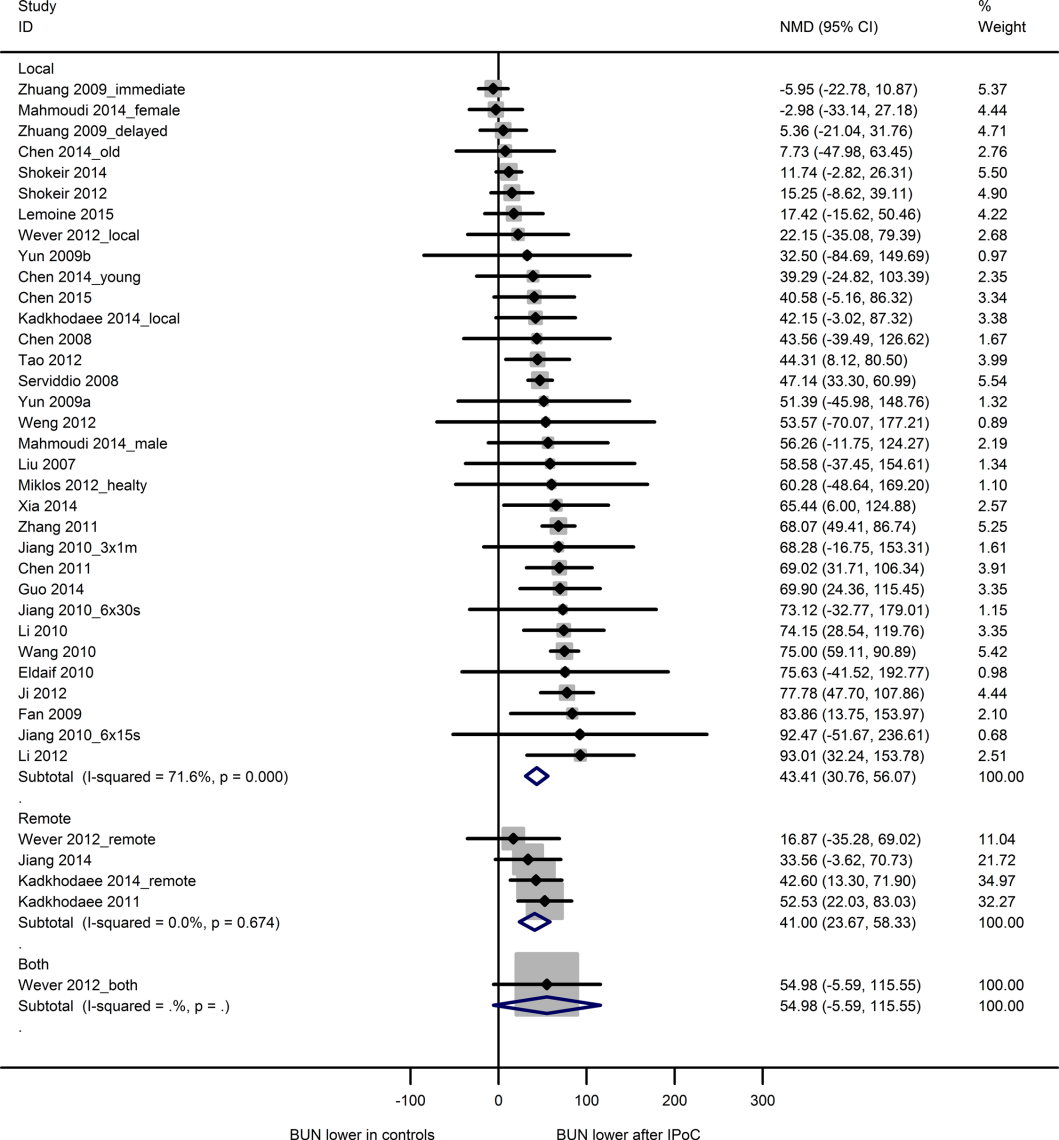
Meta-analysis blood urea nitrogen. The summary effects show a decrease in blood urea nitrogen after local or remote IPoC. One study investigating the combination of local and remote IPoC showed no effect. Data are presented as NMD and 95% CI. Within subgroup weights from random effects analysis are shown.

Twenty-eight studies reported BUN data from 36 experiments, using 226 sham animals, 222 control animals and 269 IPoC-treated animals. Both the control and experimental groups contained 3 to 12 animals (median n = 8). The IRI-induced rise in BUN was reduced by both LIPoC (33 experiments; NMD 43.4 [30.8, 56.1]) and RIPoC (4 experiments; NMD 41.0 [23.7, 58.3]; Fig 4). One study investigating the combination of LIPoC and RIPoC showed no effect (NMD 55.0 [-5.6, 115.6]).

Subgroup analysis results for the LIPoC studies are shown in S4 Table. The effect of species and sex on LIPoC efficacy could not be analyzed due to insufficient data. LIPoC had a beneficial effect on BUN in all subgroups, except for mouse, female, 4 cycles of IPoC, 631–3162 seconds of IPoC ischemia and 16–30 minutes of index ischemia. Overall heterogeneity was high (I^2^ 71.6%). A significant proportion of heterogeneity was explained by the duration of index ischemia (adjusted R^2^ 44.5%; p<0.007), indicating that the efficacy of postconditioning increased with the duration of index ischemia. None of the other subgroup variables accounted for a significant proportion of heterogeneity.

#### Renal histology

**Fig 5:**
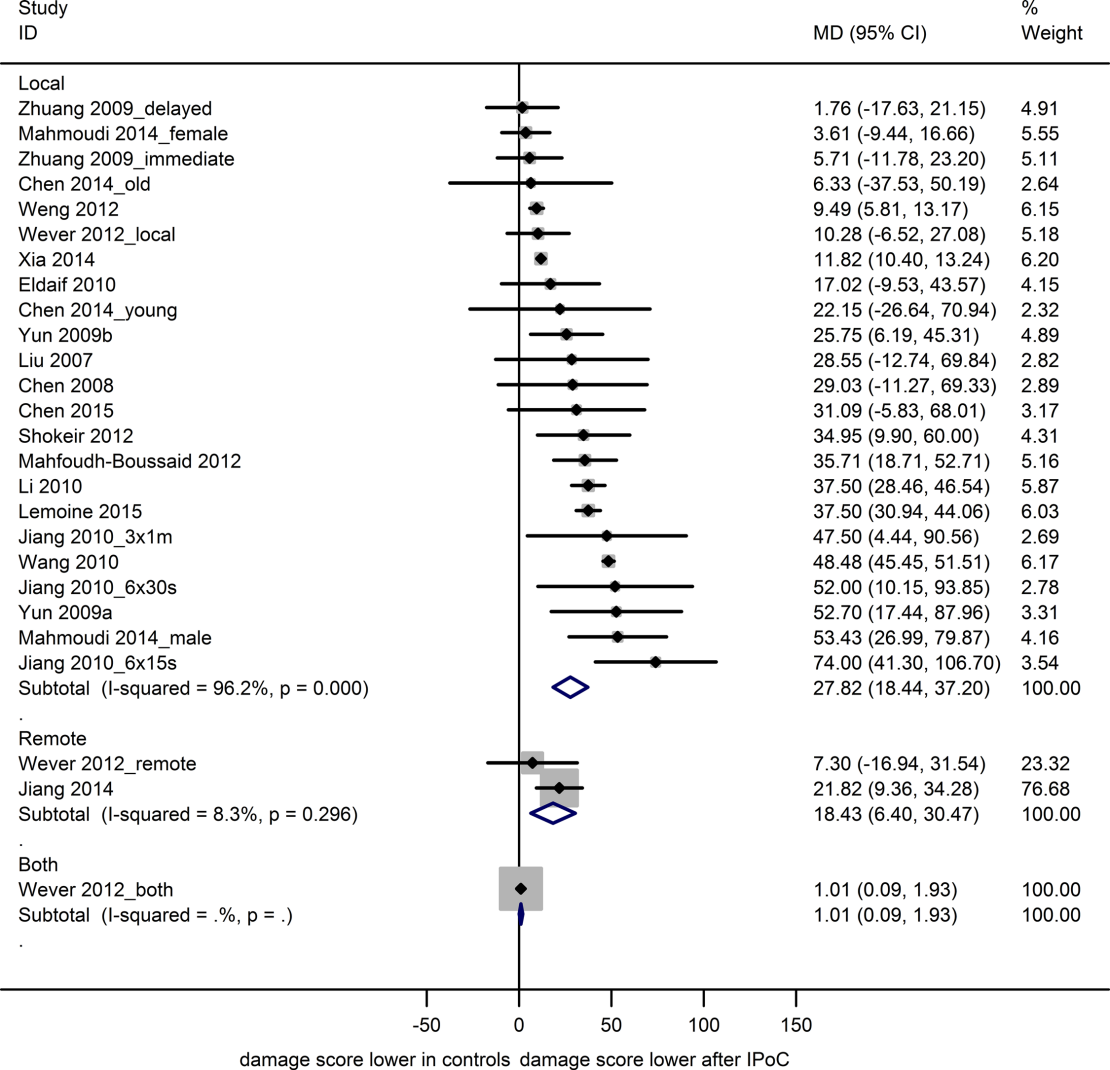
Meta-analysis renal histology. The summary effects show a decrease renal damage score after local or remote IPoC, and the combination of the two. Data are presented as MD and 95% CI. Within subgroup weights from random effects analysis are shown.

Nineteen studies reported data on renal histology from 26 experiments, using 149 sham, 152 control and 191 IPoC-treated animals. Both the control and experimental groups contained 4 to 10 animals (median n = 8). Renal histology scores were reduced after renal IRI in animals treated with LIPoC (23 experiments; MD 27.8 [18.4, 37.2] or RIPoC (2 experiments; MD 18.4 [6.4, 30.5]) or the combination of the two (1 experiment; MD 1.0 [0.1, 1.93]; Fig 5).

A positive effect of LIPoC on histology scores (S5 Table) was observed in most subgroups, similar to the results obtained for serum creatinine. The effect of species, sex and site of postconditioning on IPoC efficacy could not be analyzed due to insufficient data. Overall heterogeneity was very high (I^2^ 96.2%), but could not be attributed to any of the subgroup variables.

### Publication bias

**Fig 6:**
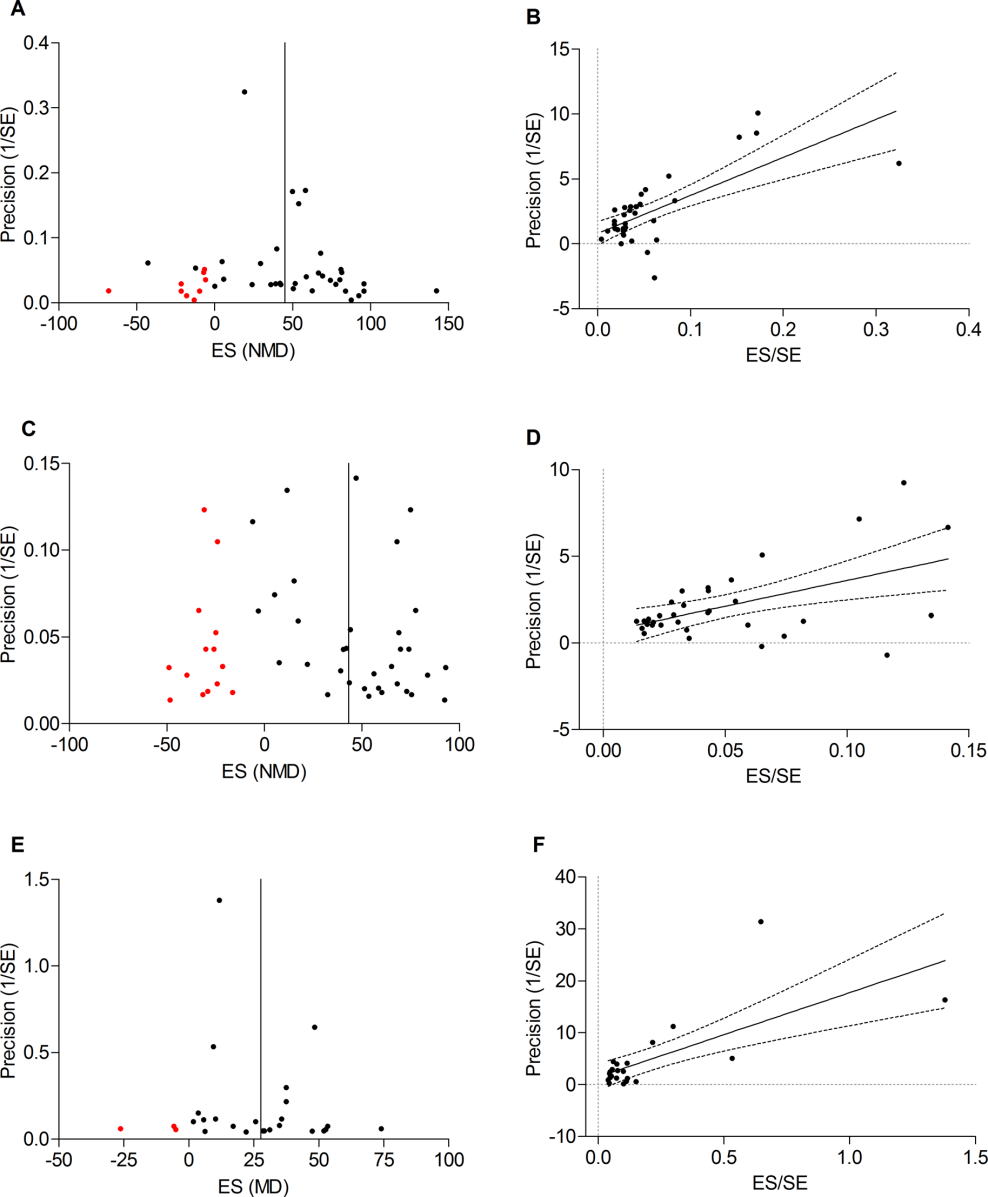
Publication bias. Trim and fill analysis for studies on local IPoC indicates funnel plot asymmetry for respectively creatinine (A), BUN (C) and renal histology (E). The 95% confidence interval of Egger’s regression line (dashed lines) does not include the origin of the graph, indicating no small study effects for creatinine (B), BUN (D) and renal histology (F).

Publication bias could be assessed for LIPoC only, due to insufficient data for RIPoC. Possible publication bias was observed for all outcome measures when visually evaluating funnel plots for asymmetry. Duval and Tweedie’s trim and fill analysis resulted in filled data points for all outcome measures (Fig 6A, 6C and 6E), indicating that small, negative studies were underrepresented. However, Egger’s test indicated that no small study effects were present (Fig 6B, 6D and 6F).

### Sensitivity analyses

Sensitivity analyses were performed to assess the robustness of our findings. For serum creatinine and BUN, a fixed time point of 24 hrs for outcome assessment was chosen instead of the time point of the greatest efficacy. The analyses contained 24 studies for both creatinine and BUN. The summary effect found in the sensitivity analysis did not differ from the original analysis for either creatinine (NMD 43.3 [30.7, 55.9] *versus* 45.0 [33.4, 56.6]) or BUN (NMD 37.3 [24.7, 50.8] *versus* 43.4 [30.8, 56.1]). The overall heterogeneity was slightly lower in the sensitivity analyses (I^2^ 64.3% *versus* 74.7% for creatinine and 69.3% *versus* 71.6% for BUN).

For renal histology, a sensitivity analysis was performed by excluding all studies which did not use the Jablonski grading scale. The summary effect found in the sensitivity analysis on the remaining 13 studies did not differ from the original analysis (MD 40.2 [32.5, 47.8] versus 27.8 [18.4, 37.2]). However, heterogeneity was considerably lower in the sensitivity analysis (I^2^ 41.5% vs. 96.2%). This was surprising, since all scales roughly scored the same features of tubular damage (*e*.*g*. cellular vacuolization, loss of brush border, cast formation). However, since the overall effect of IPoC was robust, we feel that our decision to pool all scoring systems is justified.

One study[28] measured serum creatinine and BUN twelve weeks after renal IRI. At this time-point, values were similar in all groups, which resulted in extremely large confidence intervals in our NMD meta-analysis. However, omitting this study had no effect on meta-analysis outcomes.

## Discussion

### IPoC efficacy and sources of heterogeneity

This systematic review and meta-analysis provides a quantitative summary of all preclinical *in vivo* evidence on IPoC against renal IRI. Our review shows a protective effect of both LIPoC and RIPoC on renal function and histology, based on a reduction in serum creatinine, BUN and renal histology scores. The high between-study heterogeneity was partially explained by the duration of index ischemia, *i*.*e*. LIPoC efficacy appeared to increase as the duration of index ischemia increased. The other study characteristics under investigation did not account for significant proportions of heterogeneity, or could not be analysed due to insufficient data (especially for RIPoC). For LIPoC, the remaining heterogeneity is high, especially for renal histology. Importantly, differences in the risk of bias between studies may represent a significant source of unexplained heterogeneity, but insufficient reporting currently prevents us from testing this hypothesis (see below).

### Methodological quality

Adequate reporting of methodological details is crucial to determine the risk of bias in primary studies and to assess the quality of a body of evidence. Insufficient reporting of preclinical research methodology occurs in many fields and is often associated with an overestimation of treatment effects ^e.g.^[29–31]. We show that details on key measures to reduce bias (such as randomisation and blinding) and other study quality indicators were missing from many studies included in our review. The risk of bias in most studies therefore remains unclear. Consequently, some studies may have overestimated the effect of IPoC, which may have influenced the outcome of our meta-analysis.

The number of animals per group was very low in a number of studies. This is a matter of concern, since underpowered studies have an increased risk of finding false positive results. Systematic reviews have suggested that underpowering of *in vivo* studies is common, and that this greatly contributes to translational failure[31,32]. Since none of the included studies reported a sample size calculation, we cannot exclude the possibility of an effect of underpowering on our meta-analysis.

### Study characteristics

The present review points out several apparent differences between the experimental design of current clinical trials on renal IPoC, and the preceding animal studies. Firstly, we show that 99% of the preclinical evidence was obtained from animals undergoing warm renal ischemia. In contrast, the two published clinical trials on IPoC[7,8], as well as a third trial in progress (ISRCTN66437627), all study the effect of IPoC after renal transplantation. To our knowledge, these trials were predominantly based on results obtained in animal models of warm IRI. Only one animal study investigating IPoC after renal transplantation[33] was retrieved by our search (which was not limited to a specific model of renal IRI). This study does show a protective effect of local IPoC after transplantation, however, there are substantial differences between these models (*e*.*g*. warm *versus* cold ischemia and renal denervation), and animal models using warm ischemia may not optimally predict outcomes in the clinical transplantation setting.

Secondly, we show that 90% of the animal studies investigated LIPoC, even though RIPoC is generally considered to be more applicable in clinical practice. Thus far, one clinical trial investigated LIPoC[7], and two applied RIPoC ([8] and ISRCTN66437627). Regarding the LIPoC protocol, our meta-analysis did not identify any factors related to timing or duration which influence its efficacy. Since nearly all evidence was obtained in rats, it remains unclear whether the same timing and duration is effective in all species (including humans). Only two studies have used larger animals, whose metabolic rate is more comparable to humans. Of note, Van den Akker et al [7] adjusted their clinical IPoC protocol to fit the metabolic rate in humans, but found no beneficial effect. Furthermore, there is not enough preclinical evidence to assess if timing and/or duration of the protocol influences the efficacy of RIPoC. We suggest that the optimal timing and duration of the postconditioning protocols should be determined separately for LIPoC and RIPoC.

Concerning the population under investigation, nearly all preclinical studies used male animals, whereas the clinical trials included both men and women. This sex bias (which is widespread in preclinical studies) is reason for concern, considering the evidence that females react differently to both IRI[34] and IPoC[35]. Secondly, we found no studies using animals with relevant comorbidities such as hypertension or diabetes mellitus, which are often present in patients undergoing renal surgery or transplantation. The absence of comorbidities in experimental animals has previously been described as a possible explanation for the translational failure of conditioning strategies [36–38].

### Publication bias

Visual inspection of funnel plots, as well as trim and fill analysis, indicate a possible presence of publication bias in this field. The direction of effect did not change after trim and fill, but neutral and negative studies were underrepresented. On the other hand, Egger’s test did not indicate any small-study effects, and Funnel plot asymmetry may be explained by other factors such as true heterogeneity, study quality or chance[39]. Based on this analysis we assess the risk of publication bias to be mild (histology and creatinine) to moderate (BUN). This should be kept in mind when interpreting our results, since data from a range of animal studies strongly suggested that publication bias is associated with a substantial overestimation of treatment effects[40].

### Clinical implications and future perspective

This review is the first systematic overview of preclinical evidence for the efficacy of IPoC in animal models of renal IRI. It provides useful insights in the variables influencing IPoC efficacy, within the limitations inherent to combining data from different experiments. Sensitivity analyses showed that the observed overall efficacy is robust for all outcome measures. Our finding that IPoC efficacy may increase with the duration of renal ischemia suggests that IPoC is less effective when kidney injury is mild. The severity of renal IRI in patients varies with the type of surgery they receive, their co-morbidities and additional measures which can be taken to reduce IRI. Thus, IPoC may not be equally potent in all patients, and this should be taken into account when including patients in clinical trials and analyzing clinical and preclinical results.

We also find that the body of evidence on which clinical trials are presently based is narrow, and its quality unclear. In particular, indirectness and risk of bias are reasons to interpret the preclinical findings with care. The present review points out a number of opportunities for improvement and future research, in order to increase clinical relevance of the preclinical studies and provide sufficient validity to guide clinical trial design. Preclinical studies should use both sexes, animals with relevant comorbidities, and it should be investigated whether the results obtained thus far can be replicated in transplantation models. Larger animal species may be used to better resemble the metabolic rate in humans. Importantly, to avoid effects of insufficient reporting, underpowering and publication bias in systematic reviews, it is of the utmost importance that the design, execution and reporting of animal studies is improved, for instance through the use of the GSPC and ARRIVE guidelines by authors and journals. Only then, preclinical evidence can be used to its full extent.

## Supporting Information

S1 Text Extended materials and methods. (PDF)

S1 Table Full search strategy. (PDF)

S2 Table Study quality and risk of bias assessment, individual scores. (PDF)

S3 Table Subgroup analyses serum creatinine local IPoC. (PDF)

S4 Table Subgroup analyses blood urea nitrogen local IPoC. (PDF)

S5 Table Subgroup analyses renal histology local IPoC. (PDF)

S6 Table PRISMA checklist. (PDF)

## Abbreviations

AKI: acute kidney injury
BUN: blood urea nitrogen
CI: confidence interval
IPoC: ischemic postconditioning
IRI: ischemia-reperfusion injury
LIPoC: local ischemic postconditioning
MD: raw mean difference
NMD: normalised mean difference
RIPoC: remote ischemic postconditioning
SMD: standardised mean difference
