# Supplementary material for: Preclinical Evidence for the Efficacy of Ischemic Postconditioning against Renal Ischemia-Reperfusion Injury, a Systematic Review and Meta-Analysis
Source: PLoS One. 2016 Mar 10;11(3):e0150863. doi: 10.1371/journal.pone.0150863 (PMC4786316; doi:10.1371/journal.pone.0150863)
Supplement: S1 Text — (PDF) [file pone.0150863.s001.pdf]

S1 Text – Extended materials and methods

The review methodology was predefined and documented in a systematic review protocol[15], published online on February 12th 2015. The review question was: what is the effect of local or remote IPoC on renal function in animal models of renal IRI?

2.1 Amendments to the review protocol

After study selection, we found that the timing and duration of the IPoC protocol depended strongly on the site of postconditioning. We therefore decided to perform separate meta-analyses of studies using local, remote, and local+remote postconditioning, to avoid collinearity.

For serum creatinine and blood urea nitrogen (BUN), all data could be expressed in the same unit of measurement, but differences in baseline measurements between studies were observed. We therefore performed meta-analysis of the normalised mean difference (NMD) instead of the standardized mean difference (SMD). For renal histology, we expressed all scores as a percentage on the grading scale used, and performed meta-analysis of the mean difference (MD), instead of the SMD. This allowed us to include studies reporting the histology score as a percentage on the grading scale used.

2.2 Study identification

A systematic, computerized search in the databases Medline (via PubMed) and EMBASE (Supplemental table S1) was performed on February 4th 2015, using the search components 'kidney', 'ischemic postconditioning' and an animal search filter for either PubMed[16] or EMBASE[17]. To identify additional relevant studies, the reference lists of included studies and relevant reviews were hand searched. No language restrictions were applied. Studies in a language other than English were translated using Google Translate. In case of uncertainties, a native speaker of the language was consulted.

2.3 Selection of studies

After removal of duplicates, selection of studies was performed using Early Review Organizing Software (Institute of Clinical Effectiveness and Health Policy, Buenos Aires, Argentina). All references were first screened for inclusion based on their title and abstract. The following inclusion criteria were applied: the study 1) is an original article presenting unique data with a control group, 2) is performed *in vivo* in animals with or without comorbidities, but without genetic modifications, 3) reports on renal ischemia-reperfusion injury and outcome measures related to kidney injury or function, and 4) examined the effect of remote and/or local ischemic postconditioning. Subsequently, the full-text manuscripts of eligible studies were reviewed for inclusion. Studies involving co-medication other than anaesthetics or analgesics, or a co-intervention other than collateral nephrectomy (*e.g.* renal transplantation) were excluded. In both phases, references were independently assessed for inclusion by two reviewers (KW and SJ). In case of discrepancies, consensus was reached through discussion. Authors of eligible conference abstracts were contacted through e-mail in order to retrieve the full manuscript if available. If there was no reply within three weeks after sending a reminder, the study was excluded from analysis.

2.4 Study characteristics and data extraction

The following study characteristics were extracted: bibliographical data (author, year, title, language), animal characteristics (species, strain, sex, age, weight), experimental groups, number of animals per group, duration of index ischemia, and details of the IPoC protocol (site of IPoC, number of cycles, duration of ischemia and reperfusion). One reviewer extracted the data (SJ) and a second reviewer (TM) checked the data for inconsistencies.

Based on their clinical relevance, we selected the following outcome measures for analysis: serum creatinine, BUN and renal histology scores (Jablonski[18] or comparable). Data was collected as mean and standard deviation (SD). For serum creatinine and BUN, all data was recalculated to the

same unit of measurement (umol/L for creatinine and mmol/L for BUN). For renal histology, scores were expressed as a percentage on the grading scale used. If an outcome was measured at several time-points, data was extracted for the time-point of greatest efficacy. If a study reported data from several experimental groups, it was extracted as separate comparisons and the number of animals in the control group was corrected (number of animals divided by number of comparisons). If data was only presented graphically, it was extracted using digital imaging software (ImageJ, National Institutes of Health, USA). Authors were contacted to provide additional information in case of unreported or unclear data. If there was no reply within three weeks after sending a reminder, a conservative estimate was made.

2.5 Risk of bias and study quality

Two reviewers (SJ and TM) independently assessed the risk of bias and study quality of each included study. In case of discrepancies, consensus was reached by discussion with a third reviewer (KW). Risk of bias was assessed using SYRCLE's Risk of Bias tool[19]. Reporting bias (item #9) was not assessed, since none of the studies reported the use of a study protocol predefining primary and secondary outcomes. When assessing selection bias, groups within a study were considered similar at baseline if sex and baseline serum creatinine did not significantly differ between groups (or, if baseline creatinine was unavailable, body weight). To assess whether studies were free of other risks of bias, addition of animals to groups during the experiment and a possible conflict of interest were taken into account. We also assessed reporting of the following study quality items: any randomization, any blinding, regulation of body temperature within 3°C variation, a sample size calculation and a conflict of interest statement.

2.6 Data analysis

Data was analyzed using Stata/SE (StataCorp, Texas, USA). For the outcome measures serum creatinine and BUN, meta-analysis was performed on the NMD, which allows us to correct for baseline kidney injury by relating the magnitude of the effect of treatment to a baseline measured in untreated animals[20]. For histology, the MD was used. A random effects model was used to account for expected between-study heterogeneity. To assess heterogeneity, the I^2 and adjusted R^2 statistics were determined. To examine potential sources of heterogeneity, predefined subgroup analyses were performed on subgroups containing data from at least three studies. For the duration of IPoC ischemia, studies were categorized using increments of 0.7 log, which resulted in categories of 26-125, 126-630 and 631-3162 seconds of ischemia. For the duration of index ischemia, studies were categorized using increments of 15 minutes, resulting in categories of 16-30, 31-45, 46-60, 61-75 (no studies) and 76-90 minutes. Differences between subgroups were determined by calculating the difference in NMD and MD respectively and the 95% confidence intervals (CI) of the difference. Results are reported as a NMD or MD [95%-CI], unless stated otherwise. For each outcome measure, the significance level for subgroup analyses was adjusted for the number of analyses using the Bonferroni-Holm method[21].

Publication bias was assessed for each outcome measure by visual evaluation of funnel plots, Duval and Tweedie's trim and fill analysis and by performing Egger's test for small study effects. Sensitivity analyses were carried out for creatinine and BUN using a fixed time point of outcome assessment (24 hrs). For histology, a sensitivity analysis was performed using only Jablonski histology scores.
