# Supplementary material for: Preclinical Evidence for the Efficacy of Ischemic Postconditioning against Renal Ischemia-Reperfusion Injury, a Systematic Review and Meta-Analysis
Source: PLoS One. 2016 Mar 10;11(3):e0150863. doi: 10.1371/journal.pone.0150863 (PMC4786316; doi:10.1371/journal.pone.0150863)
Supplement: S1 Table — (PDF) [file pone.0150863.s002.pdf]

S1 Table| complete search strategy for Medline (via PubMed) and EMBASE

Medline (via PubMed)	Kidney	"kidney"[MeSH Terms] OR "acute kidney injury"[MeSH Terms] OR "kidney transplantation"[MeSH Terms] OR "nephrology"[MeSH Terms] OR "kidney"[Tiab] OR "kidneys"[Tiab] OR "renal"[Tiab] OR "nephrology"[Tiab]
	Postconditioning	"ischemic postconditioning"[MeSH Terms] OR "postconditioning"[tiab] OR "post conditioning"[tiab] OR "post-conditioning"[tiab] OR "IPostC"[tiab] OR "RIPostC"[tiab] OR “IPOC”[tiab] OR “RIPOC”[tiab] OR “IPC”[tiab] OR “RIPC”[tiab] OR “postcon”[tiab] OR "brief ischemia"[tiab] OR "brief ischaemia"[tiab] OR "transient ischaemia"[tiab] OR "transient ischemia"[tiab] OR "intermittent ischaemia"[tiab]OR "intermittent ischemia"[tiab] OR “continuous ischemia"[tiab] OR "continuous ischaemia"[tiab] OR “IPost” [tiab] OR “RIPost” [tiab] OR “rPostC” [tiab] OR “PostC” [tiab]
	Animals	Laboratory animal search filter [16]

February 4th 2015: 213 hits
NB: adding additional abbreviations of ischemic postconditioning “POC”[tiab] OR “IPO”[tiab] did not generate additional relevant hits

EMBASE	Kidney	exp kidney/ or exp acute kidney failure/ or exp kidney transplantation/ or exp kidney allograft rejection/ or (renal or kidney or kidneys or nephrology).ti,ab.
	Postconditioning	ischemic postconditioning/ or (brief ischemia or brief ischaemia or postconditioning or post conditioning or post-conditioning or transient ischaemia or transient ischemia or intermittent ischaemia or intermittent ischemia or continuous ischemia or continuous ischaemia or IPost or RIPost or IPOC or RIPOC or IPostC or RIPostC or IPC or RIPC or postcon or rPostC or PostC).ti,ab.
	Animals	Laboratory animal search filter [17]

February 4th 2015: 272 hits
