# Supplementary material for: Preclinical Evidence for the Efficacy of Ischemic Postconditioning against Renal Ischemia-Reperfusion Injury, a Systematic Review and Meta-Analysis
Source: PLoS One. 2016 Mar 10;11(3):e0150863. doi: 10.1371/journal.pone.0150863 (PMC4786316; doi:10.1371/journal.pone.0150863)
Supplement: S2 Table — (PDF) [file pone.0150863.s003.pdf]

S2 Table | study quality and risk of bias assessment, individual scores

	Reporting						Risk of bias								
	Any randomization	Any blinding	Sample size calculation	Conflict of interest statement	Temperature regulation	Overall (times Y out of 5)	Random group allocation (selection)	Groups similar at baseline (selection)	Blinded group allocation (selection)	Random housing (performance)	Blinded interventions (performance)	Random outcome ass. (detection)	Blinded outcome ass. (detection)	Reporting of drop-outs (attrition)	Other biases
Chen 2008 [41]	Y	Y[†]	N	N	Y	3	?	?[#]	?	?	?	?	?	?	?
Chen 2011 [42]	Y	N	N	N	N	1	?	?[#]	?	?	?	?	?	?	?
Chen 2014 [43]	Y	Y[†]	N	N	Y	3	?	?[#]	?	?	?	?	?	?	?
Chen 2015 [44]	Y	Y[†]	N	Y	Y	4	?	?[#]	?	?	?	?	?	H	L
Eldaif 2010 [45]	N	Y[†]	N	N	Y	2	?	?[#]	?	?	?	?	?	H	?
Fan 2009 [46]	Y	N	N	N	N	1	?	?[#]	?	?	?	?	?	L	?
Guo 2014 [47]	N	N	N	N	N	0	?	?[#]	?	?	?	?	?	?	?
Ji 2012 [48]	Y	N	N	N	Y	2	?	?[#]	?	?	?	?	?	L	?
Jiang 2010 [49]	Y	Y[†]	N	N	Y	3	?	?[#]	?	?	?	?	?	L	?
Jiang 2014 [50]	Y	N	N	Y	N	2	?	?[#]	?	?	?	?	?	L	L
Kadkhodaee 2011 [22]	Y	N	N	Y	N	2	?	?[#]	?	?	?	?	?	?	L
Kadkhodaee 2014 [51]	Y	N	N	Y	Y	3	?	?[#]	?	?	?	?	?	?	L
Lemoine 2015 [52]	N	Y[†]	N	Y	Y	3	?	?[#]	?	?	?	?	?	?	L
Li 2010 [53]	Y	N	N	N	Y	2	?	?[#]	?	?	?	?	?	?	?
Li 2012 [54]	Y	N	N	N	N	1	?	?[#]	?	?	?	?	?	L	?
Liu 2007 [55]	N	Y[†]	N	N	Y	2	?	?[#]	?	?	?	?	?	?	?
Mahfoudh-Boussaid 2012 [24]	Y	Y[†]	N	Y	Y	4	?	?[#]	?	?	?	?	?	L	L
Mahmoudi 2014 [35]	Y	N	N	N	N	1	?	?[#]	?	?	?	?	?	?	?
Miklós 2012 [26]	N	Y[†]	N	N	N	1	?	L	?	?	?	?	?	?	?
Serviddio 2008 [27]	N	Y[†]	N	Y	N	2	?	L	?	?	?	?	?	?	L
Shokeir 2012 [56]	Y	N	N	Y	N	2	?	L	?	?	?	?	?	?	L
Shokeir 2014 [57]	Y	N	N	Y	N	2	?	L	?	?	?	?	?	L	L
Szwarc 2007 [58]	N	N	N	N	Y	1	?	L	?	?	?	?	?	L	?
Tan 2013 [59]	N	N	N	Y	Y	2	?	?[#]	?	?	?	?	?	?	L
Tang 2008 [60]	Y	N	N	N	N	1	?	?[#]	?	?	?	?	?	L	?
Tao 2012 [61]	Y	N	N	Y	N	2	L	L	?	?	?	?	?	L	L
Wang 2010 [62]	Y	Y[†]	N	N	Y	3	?	?[#]	?	?	?	?	?	?	?
Weng 2012 [28]	Y	Y[†]	N	Y	Y	4	?	?[#]	?	?	?	?	?	L	L
Wever 2012 [23]	Y	Y[†]	N	Y	N	3	?	?[#]	?	?	?	?	?	L	L
Xia 2014 [25]	Y	N	N	Y	N	2	?	?[#]	?	?	?	?	?	L	L
Yun 2009 A [63]	Y	Y[†]	N	N	Y	3	?	?[#]	?	?	?	?	?	L	?
Yun 2009 B [64]	Y	Y[†]	N	N	Y	3	?	?[#]	?	?	?	?	?	?	?
Zhang 2011 [65]	Y	N	N	N	N	1	?	?[#]	?	?	?	?	?	L	?
Zhu 2008 [66]	Y	N	N	N	N	1	?	?[#]	?	?	?	?	?	L	?
Zhuang 2009 [67]	N	Y[†]	N	N	N	1	?	?[#]	?	?	?	?	?	?	?
Total Y/L	26	16	0	14	17		1	6	0	0	0	0	0	16	14

Y = yes, N = no, ? = unclear risk of bias, H = high risk of bias, L = low risk of bias; [†]Only blinded for histology; [#]groups were similar only for sex
