# Supplementary material for: Preclinical Evidence for the Efficacy of Ischemic Postconditioning against Renal Ischemia-Reperfusion Injury, a Systematic Review and Meta-Analysis
Source: PLoS One. 2016 Mar 10;11(3):e0150863. doi: 10.1371/journal.pone.0150863 (PMC4786316; doi:10.1371/journal.pone.0150863)
Supplement: S3 Table — (PDF) [file pone.0150863.s004.pdf]

S3 Table | Subgroup analysis serum creatinine local IPoC

	# pub	# comp	NMD	[95%CI]
all (T^2 539, I^2 74.7%)	29	34	45.0	[33.4, 56.6]
species				
P=0.14, adj. R^2 12.8%				
dog	1	3	79.3	[29.0, 129.6]
mouse	3	4	19.5	[-13.1, 52.0]
rat	25	27	47.2	[33.8, 60.5]
sex				
P=0.21, adj. R^2 10.4%				
female	3	3	20.9	[-18.3, 60.2]
male	27	31	47.8	[35.1, 60.6]
# cycles				
P=0.26, adj. R^2 8.8%				
3 cycles	10	11	39.6	[21.3, 58.0]
4 cycles	4	5	28.3	[-5.8, 62.4]
6 cycles	14	16	51.3	[31.4, 71.3]
10 cycles	2	2	86.2	[36.4, 136.0]
IPoC ischemia				
P=0.12, adj. R^2 8.7%				
26-125 sec	23	26	41.8	[27.5, 56.1]
126-630 sec	4	5	82.0	[45.4, 118.5]
631-3162 sec	3	3	35.9	[5.6, 66.3]
index ischemia				
P=0.15, adj. R^2 7.7%				
16-30 min	4	5	20.2	[-10.7, 51.1]
31-45 min	20	22	43.7	[28.2, 59.1]
46-60 min	4	6	72.3	[41.5, 103.1]
76-90 min	1	1	58.3	[7.5, 109.1]
delay (linear)				
P=0.20, adj. R^2 6.4%	30	34		

Total # comparisons = 6, corrected P<0.009; IPoC = ischemic postconditioning, pub = publications, comp = comparisons, NMD = normalized mean difference, adj. = adjusted. Protocol ischemia; amount of total ischemia time within IPoC protocol, delay; amount of delay between index ischemia and start IPoC protocol.
