# Supplementary material for: Preclinical Evidence for the Efficacy of Ischemic Postconditioning against Renal Ischemia-Reperfusion Injury, a Systematic Review and Meta-Analysis
Source: PLoS One. 2016 Mar 10;11(3):e0150863. doi: 10.1371/journal.pone.0150863 (PMC4786316; doi:10.1371/journal.pone.0150863)
Supplement: S4 Table — (PDF) [file pone.0150863.s005.pdf]

S4 Table |Subgroup analysis blood urea nitrogen local IPoC

	# pub	# comp	NMD	[95%CI]
all (T^2 703, I^2 71.6%)	28	33	43.4	[30.8, 56.1]
species				
not analyzed				
dog	1	3	74.4	[9.6, 139.3]
mouse	2	3	4.2	[-22.7, 31.0]
rat	25	27	48.3	[36.5, 60.0]
sex				
not analyzed				
female	2	2	30.6	[-15.3, 76.6]
male	27	31	44.0	[31.5, 56.5]
cycles				
P=0.05, adj. R^2 31.9%				
3 cycles	8	9	28.1	[12.0, 44.2]
4 cycles	4	5	29.5	[-2.4, 61.5]
6 cycles	15	17	56.2	[39.1, 73.4]
10 cycles	2	2	81.9	[35.4, 128.3]
protocol ischemia				
P=0.12, adj. R^2 8.5%				
26-125 sec	22	25	42.6	[29.0, 56.2]
126-630 sec	4	5	78.2	[39.1, 117.2]
631-3162 sec	3	3	25.2	[-3.2, 53.7]
index ischemia				
P=0.007, adj. R^2 44.5%				
16-30 min	3	4	6.2	[-17.4, 29.9]
31-45 min	19	21	43.1	[29.8, 56.4]
46-60 min	5	7	73.2	[48.5, 97.9]
76-90 min	1	1	47.1	[8.8, 85.5]
delay (linear)				
P=0.05, adj. R^2 14.4%	28	33		

Total # comparisons = 4, corrected P<0.012; IPoC = ischemic postconditioning, pub = publications, comp = comparisons, NMD = normalized mean difference, adj. = adjusted. Protocol ischemia; amount of total ischemia time within IPoC protocol, delay; amount of delay between index ischemia and start IPoC protocol.
