# Supplementary material for: Preclinical Evidence for the Efficacy of Ischemic Postconditioning against Renal Ischemia-Reperfusion Injury, a Systematic Review and Meta-Analysis
Source: PLoS One. 2016 Mar 10;11(3):e0150863. doi: 10.1371/journal.pone.0150863 (PMC4786316; doi:10.1371/journal.pone.0150863)
Supplement: S5 Table — (PDF) [file pone.0150863.s006.pdf]

S5 Table |Subgroup analysis renal histology local IPoC

	# pub	# comp	MD	[95%CI]
all (T^2 369, I^2 96.2%)	18	23	27.8	[18.4, 37.2]
species				
not analyzed				
dog	1	3	59.7	[31.7, 87.6]
mouse	2	3	17.3	[-1.5, 36.1]
rat	15	17	26.2	[17.3, 35.1]
sex				
not analyzed				
female	1	1	3.6	[-29.1, 36.3]
male	18	22	28.8	[20.6, 37.0]
cycles				
P=0.84, adj. R^2 -17.0%				
3 cycles	4	5	23.4	[5.5, 41.3]
4 cycles	2	3	22.2	[-1.0, 45.3]
6 cycles	12	14	29.5	[18.1, 40.9]
10 cycles	1	1	37.5	[2.7, 72.3]
protocol ischemia				
P=0.63, adj. R^2 -5.7%				
26-125 sec	15	18	25.5	[16.2, 34.7]
126-630 sec	3	4	36.1	[14.7, 57.4]
631-3162 sec	1	1	35.0	[-5.6, 75.5]
index ischemia				
P=0.13, adj. R^2 3.3%				
16-30 min	3	4	15.5	[-1.6, 32.6]
31-45 min	11	13	25.9	[14.6, 37.1]
46-60 min	4	6	39.9	[24.4, 55.3]
delay (linear)				
P=0.22, adj. R^2 2.45%	18	23		

Total # comparisons = 4, corrected P<0.012; IPoC = ischemic postconditioning, pub = publications, comp = comparisons, MD = mean difference, adj. = adjusted. Protocol ischemia; amount of total ischemia time within IPoC protocol, delay; amount of delay between index ischemia and start IPoC protocol.
